# Adoptive Transfer of Regulatory T Cells Protects against Coxsackievirus B3-Induced Cardiac Fibrosis

**DOI:** 10.1371/journal.pone.0074955

**Published:** 2013-09-04

**Authors:** Yanxia Cao, Wei Xu, Sidong Xiong

**Affiliations:** 1 Institute for Immunobiology, Shanghai Medical College, Fudan University, Shanghai, P. R. China; 2 Institutes of Biology and Medical Sciences, Soochow University, Suzhou, Jiangsu Province, P. R. China; University of Western Ontario, Canada

## Abstract

**Background:**

Cardiac fibrogenesis in the late stage of viral myocarditis causing contractile dysfunction and ventricular dilatation, is a major pathogenic factor for the progression of myocarditis to serious cardiovascular diseases including dilated cardiomyopathy (DCM) and congestive heart failure (HF). Recent studies indicate that regulatory T cells (Tregs) are involved in the fibrotic process of liver and lung fibosis. However, the role of Tregs in the development of viral myocarditis-caused cardiac fibrosis and their therapeutic potential remains unclear.

**Methodology/Principal Findings:**

Myocardial fibrosis was induced in BALB/c mice by intraperitoneal injection of Coxsackievirus B3 (CVB3) assessed by picrosirius red staining and detection of expression levels of collagen I, matrix metalloproteinase-1 (MMP-1), matrix metalloproteinase-3 (MMP-3) and tissue inhibitor of metalloproteinase-1 (TIMP-1). Myocardial Treg frequency was down-regulated during the course of viral myocarditis and a negative correlation with the severity of cardiac fibrosis was found. To explore the role of Tregs in CVB-induced cardiac fibrosis, Treg was in vivo depleted by injecting anti-CD25 mAb which resulted in aggravation of cardiac fibrosis. In consistent with that, after adoptive transfer of isolated Tregs into mice, significant amelioration of CVB3-induced cardiac fibrosis was confirmed. Interleukin-10 (IL-10) neutralizing antibodies were used in vivo and in vitro to explore the molecular mechanism of the therapeutic effect of Treg. It was found that administration of anti-IL-10 mAb after Treg transfer abrogated Treg’s treating effect and the inhibition of Treg on collagen production by cardiac fibroblasts was mediated mainly through IL-10.

**Conclusion/Significance:**

Our data suggested that Tregs have a protective role in the fibrotic process of CVB3-induced cardiac fibrosis via secreting IL-10 and might provide an alternative option for the future treatment of cardiac fibrosis.

## Introduction

Cardiac fibrosis is characterized by progressive accumulation of fibrillar extracellular matrix (ECM) proteins in the myocardium and occurs in the later stage of heart failure (HF) following cardiomyocyte hypertrophy, necrosis and apoptosis [1–3], which is the outcome of chronic inflammatory reactions induced by a variety of stimuli including injury, autoimmune reactions and persistent infections. Viral myocarditis induced by enteroviruses infection often progress to serious cardiovascular diseases including dialated cardiomayopathy (DCM) and HF [4,5], during which cardiac fibrosis is a key pathogenic factor, contributing to ventricular contractility and functionality impairment [6–8]. Coxsackievirus of B3 group (CVB3) infection is a leading cause of acute and chronic viral myocarditis and was reported to cause interstitial collagen deposition [9]. Despite extensive investigation aimed at pathogenic factors of cardiac fibrosis, the cellular and molecular factors contributed to cardiac fibrosis are not fully understood and currently no effective therapy are available for treating cardiac fibrosis.

Various innate and adaptive immune cells have been reported to be involved in the fibrotic process including inflammatory monocytes, neutrophils, macrophages and CD4^+^ Th cells [10–13]. Th2-immunity is thought as a potent driver of progressive fibrosis while Th1 mediated immune response shows anti-fibrotic activity [14,15]. Regulatory T cells (Tregs), a subset of CD4^+^ lymphocytes expressing Forkhead box protein 3 (Foxp3), are potent suppresser of numerous inflammatory response [16]. Recent studies have found that Treg might be involved in the fibrotic process, including lung fibrosis and liver fibrosis [17–19]. Various cytokines are also important drivers for chronic inflammation and ultimate fibrosis. Transforming growth factor-β (TGF-β) is extensively involved in the development of fibrosis in different organs [8,20]. Interleukin-IL-13(IL-13) is now recognized as an important mediator in allergy and most important fibrosis [21]. IL-22 is recently reported to have anti-fibrotic functions in a murine model of alcoholic liver disease [22]. IFN-γ, IL-10 and epigenetic modulators such as microRNAs also play key roles in regulating inflammation and major matrix protein collagen synthesis [23]. Anyway, it is still unclear whether Tregs and Treg-related cytokines are involved in the fibrotic process of myocardial fibrosis.

To explore whether Treg has a role in cardiac fibrosis, in this study, a murine model of cardiac fibrosis was established by infection mice of sub-lethal dose of CVB3. The frequency of Tregs and its correlation with the severity of cardiac fibrosis were first investigated. A variety of ECM components such as type-I and -III collagen, matrix metalloproteinases (MMPs) and tissue inhibitors of metalloproteinases (TIMPs), which regulate the profibrotic properties of fibroblasts were analyzed to evaluate the severity of cardiac fibrosis as well as the immunohistochemical staining of the heart tissue. Adoptive transfer and in vivo depletion of Tregs were performed to explore the role of Treg in the development of cardiac fibrosis. Treg-fibroblast co-culture and cytokine neutralization experiments were performed to explore the molecular mechanism of Tregs to protect against cardiac fibrosis.

## Methods

### Ethics Statement

All animal experiments were carried out in accordance with the Guide for the Care and Use of Laboratory Animals of Shanghai Medical College of Fudan University and were approved by the Ethical Committee of Fudan University (Permit number: FDU20110310). All efforts were made to minimize suffering in animal experiments.

### Mice and Virus

Male inbred BALB/c (H-2d) mice (4 weeks) were purchased from Slaccas Experimental animals LLC of Shanghai Slaccas (Shanghai, P. R. China). CVB3 (Nancy strain) was a gift from Professor Yingzhen Yang (Key Laboratory of Viral Heart Diseases, Zhongshan Hospital, Shanghai Medical College of Fudan University) and was maintained by passage through Hela cells (ATCC number: CCL-2). Virus titer was determined prior to infection by a 50% tissue culture infectious dose (TCID_50_) assay of Hela cells monolayer. Mice were infected by an intraperitoneal injection of 100 µl of phosphate-buffered saline (PBS) containing approximately 10 TCID_50_ of the virus.

### Quantitative real-time PCR Analysis

Total RNA was extracted from hearts or cardiac fibroblasts by Trizol reagent (Invitrogen) and was reversed into cDNA. Quantitative real-time PCR was performed to evaluate the relative expression of collagen I (forward primer: CGCCATCAAGGTCTACTGC, reverse primer: GAATCCATCGGTCATGCTCT), MMP-1 (forward primer: TGTTTGCAGAGCACTACTTG, reverse primer: CAGTCACCTCTAAGCCAAAG), MMP-3 (forward primer: AAAGACAGGCAC TTTTGGCG, reverse primer: CCCTCGTATAGCCCAGAACT), TIMP-1 (forward primer: CTTGGTTCCCTGGCGTACTC, reverse primer: ACCTGATCCGTCCAC AAACAG) and IL-10 (forward primer: GGTTGCCAAGCCTTATCGGA, reverse primer: ACCTGCTCCACTGCCTTGCT) using LightCycler DNA Master SYBR Green I Kit (Takara), normalizing by GAPDH (forward primer: CTCTGG AAAGCTGTGGCGTGATG, reverse primer: AAAGACAGGCACTTTTGGCG) expression. The number of PCR cycles (Ct) was calculated for the test and reference reactions. Ct values were treated to obtain ΔCt where ΔCt = Ct (test locus) − Ct (GAPDH). The relative DNA copy number was calculated as 2^−ΔΔCt^ where ΔΔCt = ΔCt (test DNA) − ΔCt (control DNA).

### Histopathology

Heart tissues of mice were cut longitudinally, fixed in 4% phosphate-buffered formalin, paraffin-embedded, sectioned. Histological analysis was performed on deparaffinized 5-μm-thick tissue sections, which were stained with hematoxylin/eosin (HE) to assess myocardial inflammation and with picrosirius red (Sigma) to visualize the degree of fibrosis. Stained sections were observed by Nikon Eclipse TE2000-S microscope (Nikon, Japan) and true color digital images were captured. For quantitative evaluation of fibrosis, collagen volume fraction (CVF) was calculated using Leica QWin image analysis software, showed as percentage of area fractions of fibrosis per total areas of heart tissue sections.

### Flow cytometry Analysis

Peripheral Blood Mononuclear Cell (PBMC) and myocardial infiltrated cells were collected as previously described [24]. Then, the cells were stained with PerCP-labeled anti-mouse CD4 (eBioscience) and FITC-conjugated anti-mouse CD25 (eBioscience) at 4 °C for 40 min. After washing, fixing and permeabilization, the cells were stained intracellularly with PE-conjugated anti-mouse Foxp3 (eBioscience) at 4 °C for 1 hour before by flow cytometry analysis on a FACS Calibur machine (BD Biosciences). Data were analyzed with CellQuest software (BD Biosciences).

### Isolation and adoptive transfer of CD4^+^CD25^+^ T cells

Spleens from infected mice were removed and prepared for single-cell suspensions under sterile conditions. Tregs were isolated using CD4^+^CD25^+^ Regulatory T Cells Isolation Kit (Miltenyi Biotec). Briefly, CD4^+^ T cells were pre-enriched by depletion of non-CD4^+^ T cells using a cocktail of biotin-labeled antibodies, anti-biotin magnetic beads and a LD magnetic bead column; To isolate CD4^+^CD25^+^ T cells, enriched CD4^+^ T cells were incubated with PE-labeled anti-CD25 antibody and anti-PE magnetic beads. Then CD4^+^CD25^+^ T cells were positively selected by a MS magnetic bead column. The purity of CD4^+^CD25^+^ T cells was more than 95%. Purified single-cell suspensions (1×10^6^ cells/mouse) were adoptively transferred one day before CVB3 challenge via tail vein injection.

### In vivo depletion of Tregs

WT mice were given 0.1 mg per dose per mouse of a depleting anti-CD25 antibody (PC61; BioXcell) or isotype antibody (Sigma-Aldrich) one day before CVB3 challenge, and then were given twice a week as previously described [25]. Depletion of Tregs in peripheral blood was confirmed by flow cytometry.

### Neutralization of IL-10

In vivo blockade of IL-10 was achieved using IL-10- neutralizing antibody (R&D Systems) as previously described [25]. Briefly, groups of 5 mice adoptively transferred with Tregs were infected with CVB3 24 hrs after adoptive transfer, and immediately injected with 100 μg of anti-IL-10 neutralizing antibody (R&D Systems) or an isotype antibody and repeatedly given the Abs twice a week for 4 weeks.

### Co-culture of cardiac fibroblasts and Tregs

To isolate the cardiac fibroblasts, heart tissues were removed from 5–10 mice under sterile conditions one week after CVB3 infection and dissected into 1 mm^3^ pieces. The tissues were incubated for 2 hours in PBS with 1 mg/ml collagenase II (Sigma) at 37 °C and then were seeded into culture dishes containing Dulbecco’s modified Eagle’s medium supplemented with 10% fetal bovine serum (FBS) and 1% penicillin/streptomycin. Cardiac fibroblasts between passages 2 and 6 were co-cultured with Tregs at ratios of 1:1, 1:2.5 or 1:5. Anti-IL-10 neutralizing antibody (R&D Systems) was added to the co-culture system at different concentration (0.5, 5 or 50 ng/ml). After 24 hours, cardiac fibroblasts were harvested; collagen I and TIMP-1 mRNA were analyzed by quantitative real-time PCR.

### Statistical Analysis

Quantitative data were provided as the means ± SEM. All statistical analyses were performed by using the GraphPad Prism (Version 5.0) statistical program. Data were tested with unpaired *t* test. P<0.05 was considered statistically significant.

## Results

### Coxsackievirus B3 infection resulted in cardiac fibrosis

**Figure 1:**
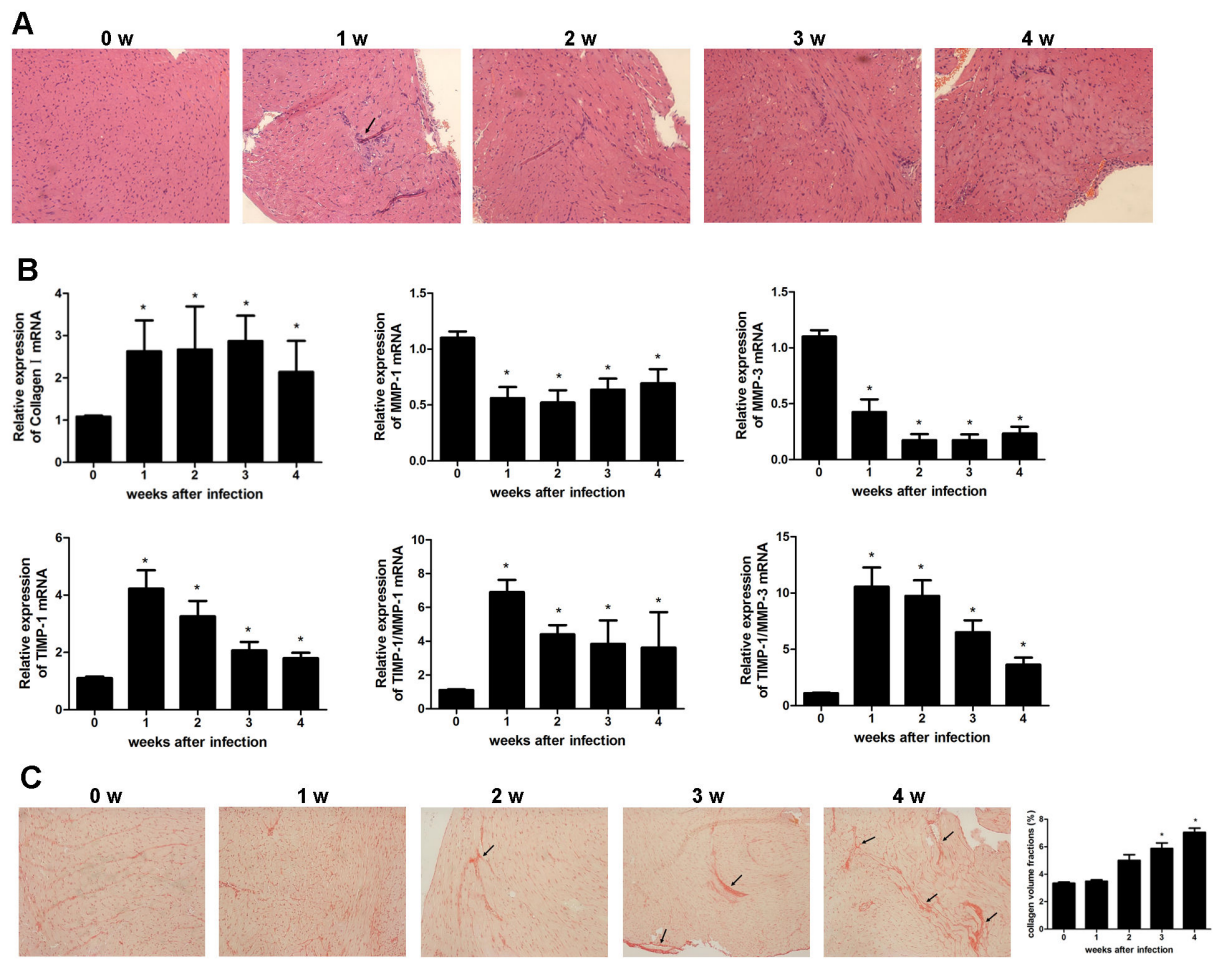
Mice have increased myocardial fibrosis after CVB3 infection. (**A**) Groups of 8 mice were infected with 10 TCID_50_ dose of CVB3, the myocardial inflammation was shown by H&E staining 4 wks after infection. Representative heart sections were shown for each week (magnification: 200×). Arrows indicate inflammatory cell infiltrate. (**B**) At 1,2,3,4 wks after CVB3 infection, the mRNA expression levels of matrix proteins (collagen I, MMP-1, MMP-3, TIMP-1) were determined by quantitative real time-PCR. Data are from one representative experiment of three performed ones and represent as the mean±SEM. (**C**) Picrosirius-red stained heart sections of CVB3-infected mice revealed increased fibrosis (red) (magnification: 200×). Arrows indicate myocardial interstitial and perivascular fibrosis. The severity of fibrosis was scored by collagen volume fractions and represent as mean ± SEM of three separate experiments. *, P<0.05 compared to the non-infected mice (wk0).

Viral myocarditis was induced in BALB/c mice 7 days after CVB3 infection showing myocardial inflammation evidenced by H&E staining (Figure 1A). To investigate whether cardiac fibrosis was also induced during the course of viral myocarditis, mRNA levels of the main product of myofibroblasts, collagen I, matrix metalloproteinases (MMPs) and tissue inhibitor of metalloproteinase-1 (TIMP-1) which regulate collagen homeostasis were examined. Collagen I mRNA was up-regulated more than two-folds in the heart tissues of infected mice compared to that in control mice **(** Figure 1B, P < 0.05), while no difference of collagen III expression was observed (date not shown). There was a significant reduction of MMP-1 and MMP-3 and an increase of TIMP-1 expression (Figure 1B, P < 0.05). The ratio of TIMP-1/MMP-1 or MMP-3 expression was significantly increased (Figure 1B, P < 0.05) indicating an increase of deposition of collagen proteins at the later stage of CVB3 myocarditis. In association with this, collagen deposition was found increased interstitially and perivascularly in myocardium of mice 4 wks after CVB3 infection (Figure 1C), evidenced by Picrosirius-red staining. The severity of fibrosis was also determined by collagen volume fraction (CVF) analysis which showed an significant increase of collagen deposition 4 weeks after infection (Figure 1C, P < 0.05). All these data suggested that cardiac fibrosis was developed in CVB3-infected mice.

### Treg frequency was negatively associated with the severity of cardiac fibrosis

**Figure 2:**
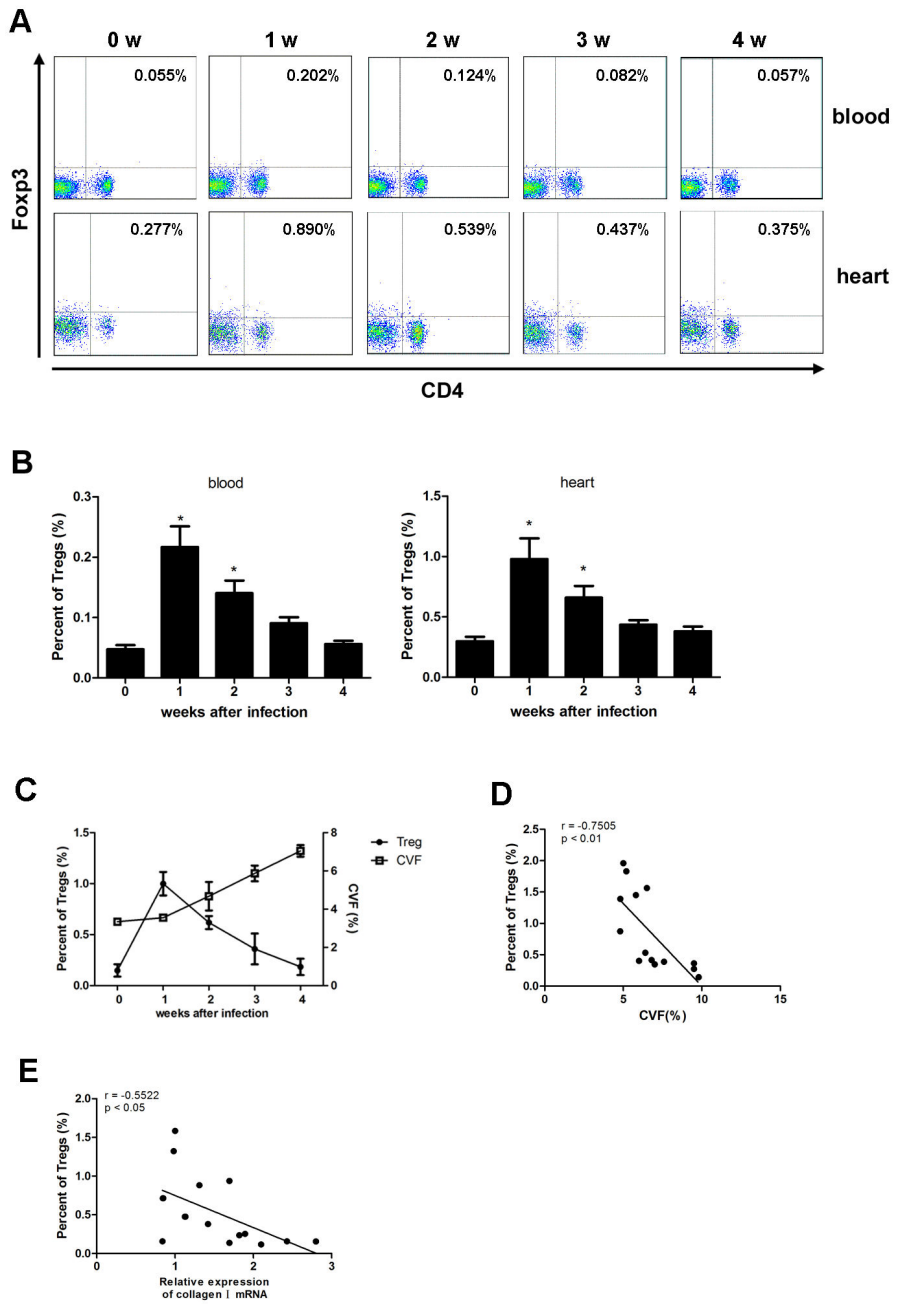
Treg frequency has negative correlation with severity of cardiac fibrosis. Groups of 20 mice were infected with 10 TCID_50_ dose of CVB3, 1,2,3,4 wks after infection, spleen and myocardial infiltrated cells were subjected to, flow cytometry protocol and stained for PerCP-CD4 and PE-Foxp3. (A) Frequencies of peripheral and myocardial Tregs were shown, graph representative of three independent experiments. (**B**) Relative numbers of Treg frequency. Data. represent as mean ±SEM. *, P<0.05 compared to non-infected mice (wk0). N=5 mice per group (**C**) Myocardial Treg frequency and the relative collagen volume fraction (CVF) of heart tissue were parallelly shown in one graph. (**D** and **E**) 4 weeks after CVB3 infection, peripheral Treg frequency and CVF or collagen I mRNA in each mouse were analyzed and correlated.

To explore the association of Tregs and severity of cardiac fibrosis, the frequencies of peripheral and myocardial Tregs after CVB3 infection was detected by flow cytometry. As shown in Figure 2A and B, Tregs were significantly up-regulated 1 wk following CVB3 infection with the relative and absolute numbers of myocardial Tregs mounting about 1% and 2×10^3^ per mouse respectively (**P<0.05**), then decreased slowly during wk2- wk 4 indicating down-regulation of Tregs at the later stage of viral myocarditis when cardiac fibrosis became evident (Figure 2C). Further, frequency of peripheral Tregs and magnitude of collagen deposition (indicated by collagen volume fraction, CVF) or collagen I mRNA in each mouse were evaluated 4 weeks after CVB3 infection. We found that mice with lower frequency of Tregs were subjected to more serious cardiac fibrosis evidenced by higher levels of CVF (Figure 2D, r=-0.7505, P<0.01) or collagen I mRNA (Figure 2E, r=-0.5522, P<0.05) indicating a negative correlation between Tregs frequency and severity of cardiac fibrosis.

### Depletion of Tregs aggravated the severity of cardiac fibrosis

**Figure 3:**
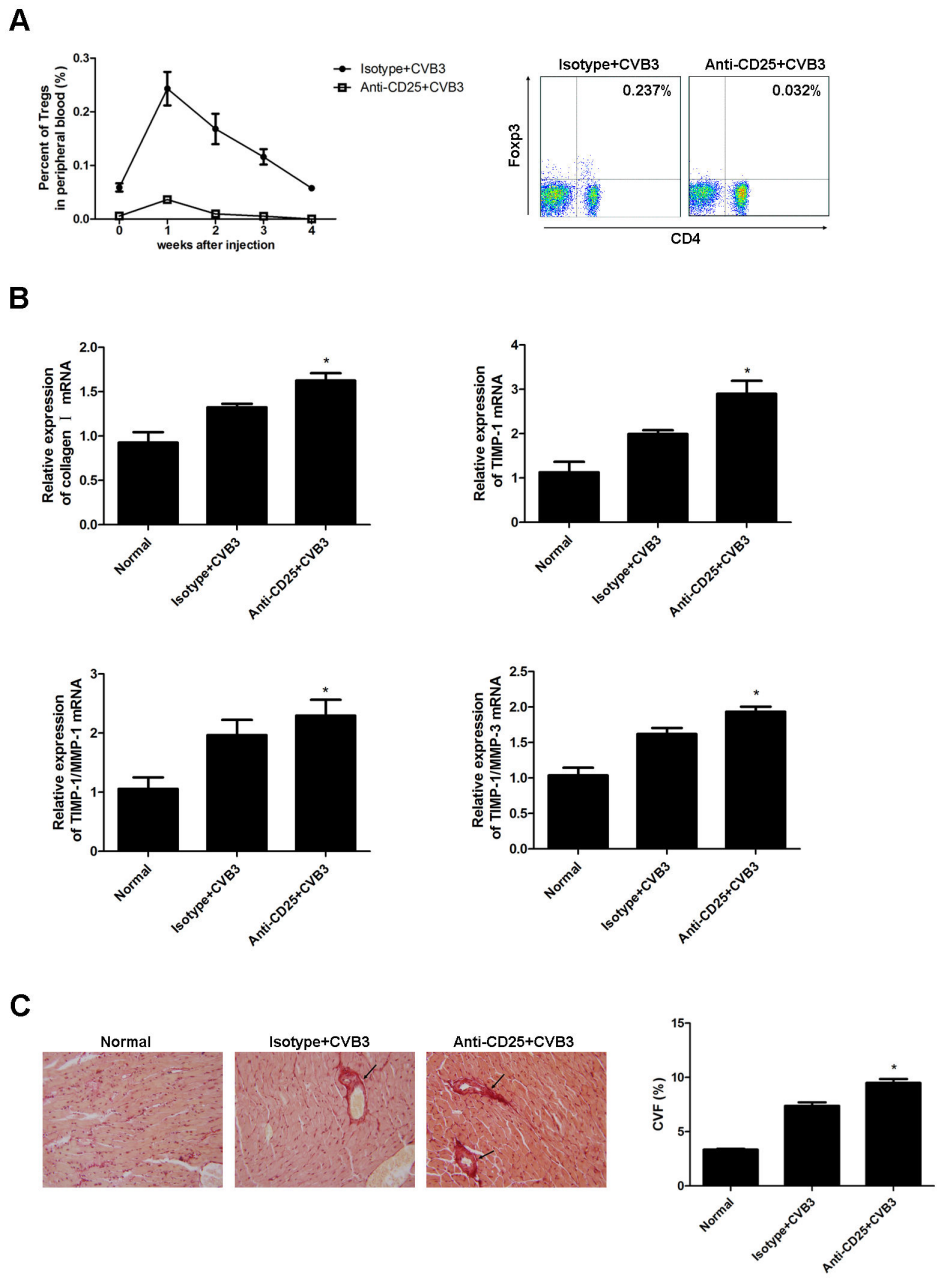
Mice Depleted of Tregs had aggravated cardiac fibrosis. (**A**) Groups of 5 mice were treated with 8 doses of 0.1 mg anti-CD25 or isotype mAb via intraperitoneal injection during the course of CVB3 infection to deplete Tregs. The peripheral Treg frequency at wk 1 was determined to confirm the depletion effect. Graph representative of three independent experiments. Relative numbers of Treg frequency are represent as the mean ±SEM. (**B**) 4 wks after CVB3 infection, mRNA levels of collagen I, TIMP-1 and relative expression of TIMP-1/MMP-1 as well as TIMP-1/MMP-3 in the heart tissues were detected. Data are from one representative experiment of three performed ones and represent as the mean ±SEM. (**C**) Representative Picrosirius-red stained heart sections (magnification: 200×) and the collagen volume fraction (CVF) at week 4. Data represent the mean ± SEM. Arrows indicate myocardial interstitial and perivascular fibrosis. Individual experiments were performed three times with similar results. *, P<0.05 compared to mice receiving isotype Ab.

Since Tregs were significantly down-regulated when cardiac fibrosis was evidenced after CVB3 infection, Treg may have a protective role in controlling cardiac fibrosis. To verify this hypothesis, depletion of Tregs was performed by intraperitoneal injection of anti-CD25 mAb one day before CVB3 infection and then twice a week, which resulted in a more than 90% reduction of peripheral Tregs (Figure 3A). The cardiac mRNA expression of collagen I, TIMP-1, TIMP-1/MMP-1 and TIMP-1/MMP-3 was significantly increased in Treg-depleted mice (Figure 3B, P < 0.05). In parallel with the data, picrosirius-red staining demonstrated aggravation of cardiac fibrosis and higher CVF in Treg-depleted mice 4 weeks after CVB3 infection compared to isotype Ab treated mice (Figure 3C, P < 0.05). These data suggested a protective role of Tregs in resolution of CVB3-induced cardiac fibrosis.

### Adoptive transfer of Tregs ameliorated cardiac fibrosis

**Figure 4:**
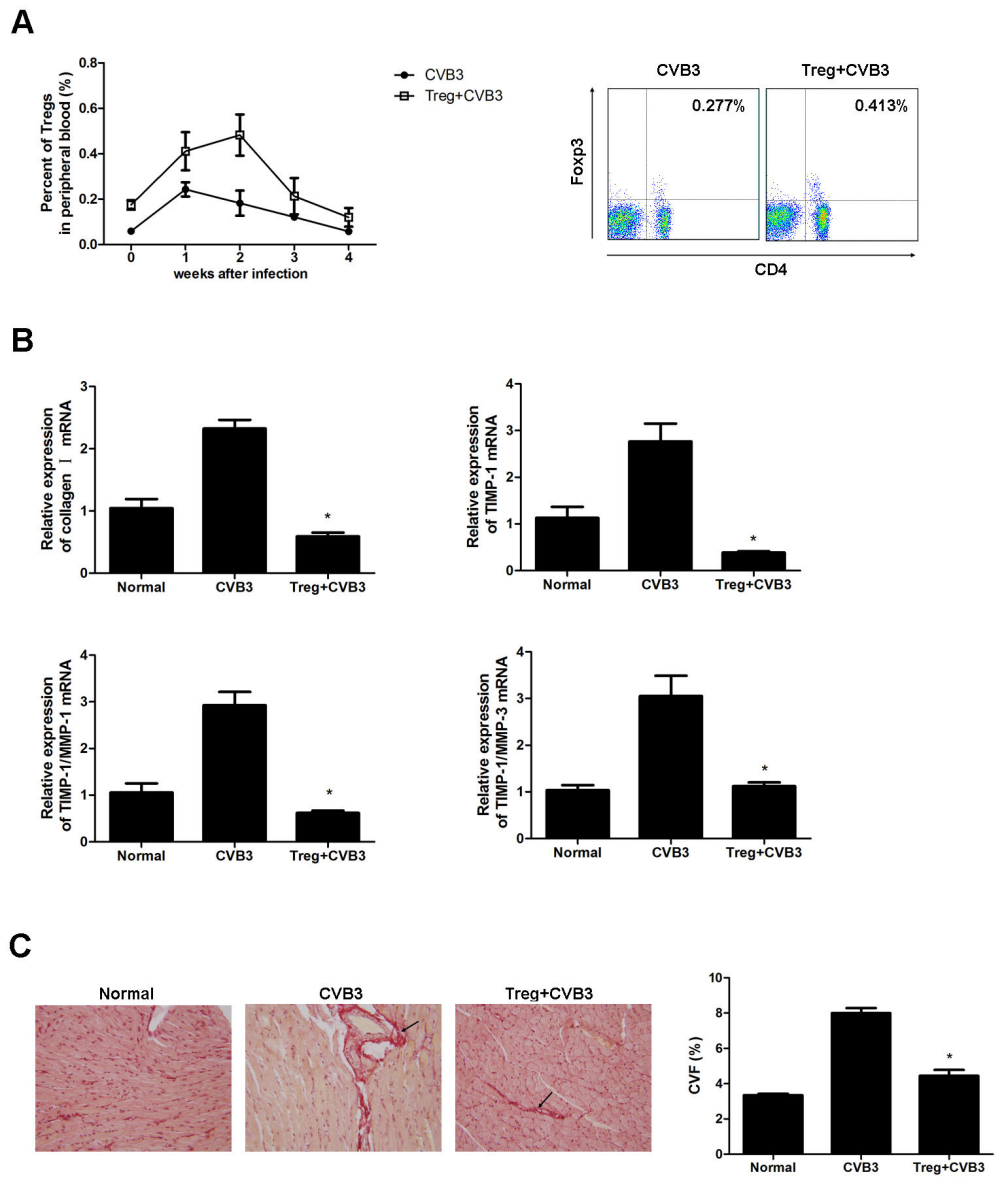
Adoptive transfer of Tregs ameliorated cardiac fibrosis in CVB3-infected mice. (**A**) Groups of 5 mice were transferred with 1×10^6^ Tregs per mouse one day before CVB3 challenge via tail vein. The peripheral Treg frequency at wk 1 was determined. Graph representative of three independent experiments. Relative numbers of Treg frequency are represent as the mean ±SEM. (**B**) RT-PCR measurement of cardiac gene expression levels of collagen I and TIMP-1, TIMP-1/MMP-1 and TIMP-1/MMP-3 4 wks after CVB3 infection. Data are from one representative experiment of three performed ones and represent the mean ±SEM. (**C**) Picrosirius-red stained heart sections at week 4 (magnification: 200×) were shown and the collagen volume fraction (CVF) was evaluated. Arrows indicate myocardial interstitial and perivascular fibrosis. Individual experiments were performed three times with similar results. *, P<0.05 compared to non-transferred mice.

To further confirm the protective role of Tregs in the development of cardiac fibrosis, splenic Tregs were isolated and adoptively transferred (AT) into mice one day before CVB3 infection. Treg frequency in the peripheral blood after adoptive transfer was confirmed by flow cytometry, showing a significant increase of peripheral Tregs compared with mock mice which lasted for 3 weeks (Figure 4A). As shown in Figure 4B, adoptive transfer of Tregs led to a significant decrease in collagen I, TIMP-1, TIMP-1/MMP-1 and TIMP-1/MMP-3 level at week 1 (**P<0.05**). And a significant amelioration of cardiac fibrosis and reduction in collagen deposition was observed in Treg-transferred mice compared to mock mice (Figure 4C, P < 0.05). The data indicated that Tregs had a protective role against CVB3-induced cardiac fibrosis.

### IL-10 was crucial for the protective role of Tregs against cardiac fibrosis

**Figure 5:**
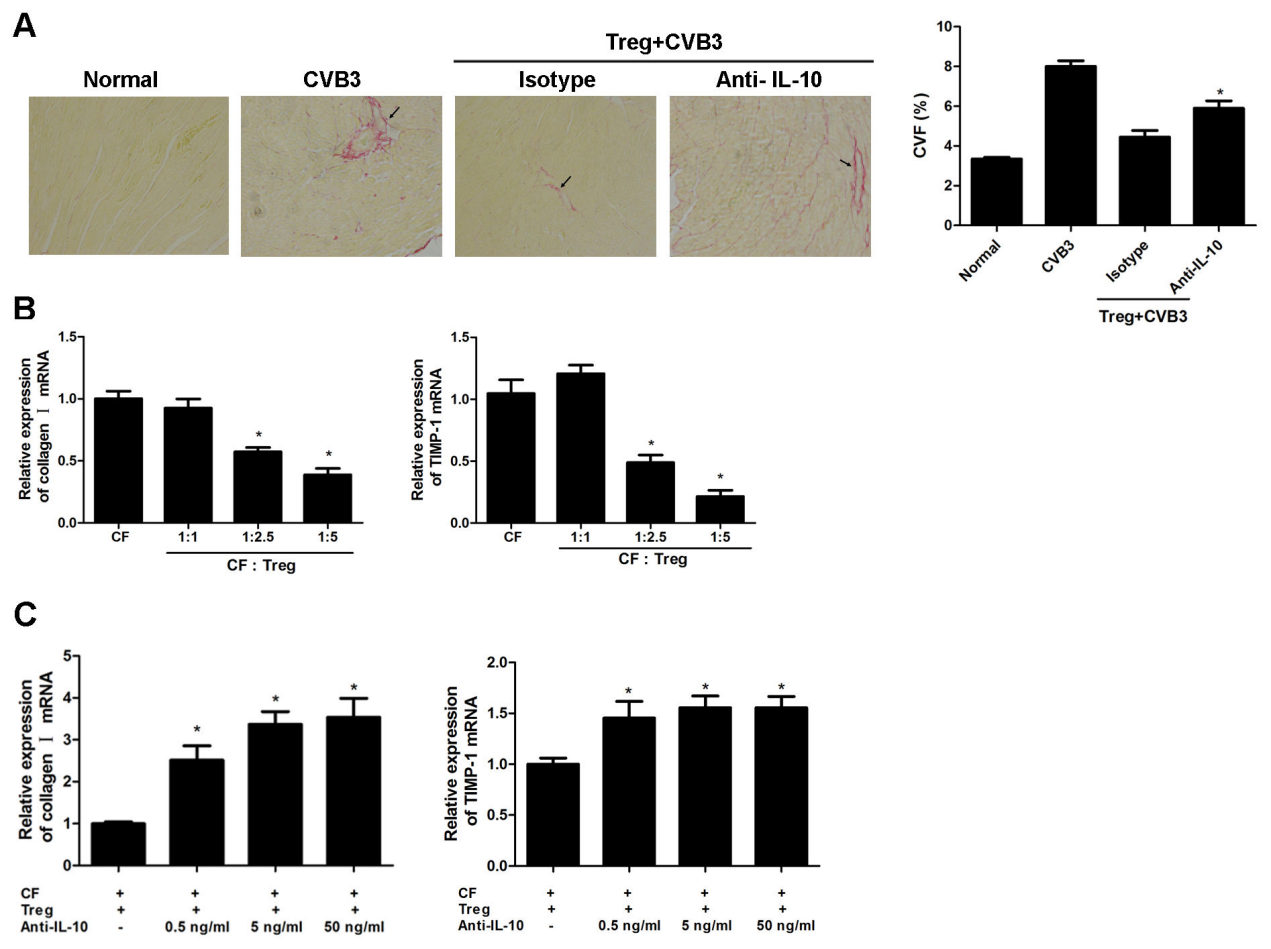
IL-10 played an important role in Tregs-mediated inhibition of cardiac fibrosis. (**A**) Groups of 10 mice were adoptively transferred with 1×10^6^ Tregs one day before CVB3 infection. 1 hr after CVB3 infection, mice were injected with 100 μg of anti-IL-10 neutralizing mAb per mouse twice a week for 4 weeks. Representative Picrosirius-red stained heart sections 4 weeks after CVB3 infection (magnification: 200×) and the collagen volume fraction (CVF) was shown. Arrows indicate myocardial interstitial and perivascular fibrosis. Data represent the mean ± SEM. *, Individual experiments were performed three times. P<0.05 compared to mice given isotype Ab after Treg transfer. (**B**) RT-PCR measurement of cardiac fibroblast gene expression levels of collagen I and TIMP-1 24 hours after isolated Tregs were co-cultured with cardiac fibroblasts (CFs) at different cell ratios (1:1, 1:2.5, 1:5). Data are from one representative experiment of three performed ones and represent as the mean ±SEM. *, P<0.05 compared to cardiac fibroblasts culture alone. (**C**) The relative expression of collagen I and TIMP-1 after anti-IL-10 mAb was added in the co-culture system. Data represent the mean ± SEM, representative of three independent experiments. *, P<0.05, compared to co-culture system with no IL-10.

The suppressive role of Tregs has been proposed to be partially mediated by IL-10. To investigate if IL-10 was involved in Treg-mediated protection against cardiac fibrosis, we treated mice with anti-IL-10 neutralizing antibody after adoptive transfer of Tregs. 4 weeks after infection, compared with CVB3-treated mice that received Tregs exhibited a significant resolution of cardiac fibrosis demonstrated by picrosirius-red staining and CVF evaluation; In contrast, the protective effect of Tregs was significantly impaired by the blockade of IL-10 (Figure 5A, P < 0.05) indicating IL-10 played an important role in Tregs-mediated protection against cardiac fibrosis. Since the progression of ECM deposition is primarily mediated by cardiac fibroblasts (CFs) by producing collagen and other matrix components, the role and mechanism of Treg in inhibiting fibrosis was further investigated in vitro by co-culturing CFs and Tregs at different ratios and collagen I, MMPs, TIMP-1 expression were determined 24 hours later. It was found that Tregs dramatically inhibited the production of collagen I and TIMP-1 by CFs in a dose-dependent manner (Figure 5B, P < 0.05). However, administration of anti-IL-10 antibody significantly abrogated the inhibitory effect of Tregs on CFs in a dose dependent manner (Figure 5C, P < 0.05) indicating that the protective role of Treg- against cardiac fibrosis was mainly mediated through secretion of IL-10.

## Discussion

Fibrosis, a pathological condition characterized by excessive synthesis and accumulation of ECM proteins in multiple organs, is associated with end-stage pathological symptoms of a wide variety of vascular injury and inflammation related diseases [23]. In the late stage of chronic viral myocarditis such as CVB3-induced myocarditis, cardiac fibrosis is a characteristic feature recognized as an important pathogenic factor contributing to serious cardiovascular diseases including DCM and HF by impairing ventricular contractility and functionality [1,9]. CD4+ Th2-type immune responses, mediated by IL-4, are believed to drive fibrotic process [14] and recent study revealed that TNF-α-Secreting B Cells may contribute to myocardial fibrosis in DCM [26]. Till now the cellular and molecular mechanism underlying myocarditis-caused cardiac fibrosis is not clear.

Tregs are important producers of immune-suppressive cytokines that control inflammatory immune response and contributes to the maintenance of self-tolerance and host homeostasis. Recent reports indicate that Tregs play important roles in the fibrotic process under inflammatory condition [17–19]. An extensive infiltration of Tregs after HCV infection was found in the liver tissue which had a negative relationship with the severity of liver fibrosis [19]. Tregs impairment was evidenced in idiopathic pulmonary fibrosis patients, which correlated with disease severity [27], suggesting a role for Tregs in the fibrotic process. So far, the role of Tregs in CVB3-induced cardiac fibrosis has not been investigated. This is the first report that Treg frequency was significantly reduced during the course of chronic viral myocarditis and was negatively correlated with the severity of cardiac fibrosis following CVB3 infection. Depletion of Tregs in CVB3-infected mice delayed the recovery of cardiac fibrosis. In accordance with that, adoptive transfer of Tregs into CVB3-infected mice was found to ameliorate the progression of cardiac fibrosis. These data are consistent with recent reports that Tregs transfer resulted in an improvement in cardiac damage and a reduction in cardiac fibrosis induced by angiotensin II [28] and Treg depletion attenuated the development of silica-induced lung fibrosis [17]. Our data provide experimental evidence that Tregs play a significant role in resolution of organ fibrosis, which provide a valuable clue for the future development of Treg-based strategies for the treatment of cardiac fibrosis.

One important molecular mechanism of Tregs to regulate immune response has been demonstrated to be mediated through production of immune-suppressive cytokines, IL-10 [29,30]. IL-10 is most commonly recognized as an immunosuppressive cytokine to control inflammation thought to be driving fibroproliferation. In our study, in vivo and in vitro experiments both demonstrated that IL-10 produced by Treg significantly contributed to the inhibition on collagen synthesis by cardiac fibroblasts. That means, IL-10 plays an antifibrotic role, which is consistent with previous reports [31,32]. And intravenous injection of IL-10-overexpressing monocytes/macrophages greatly reduced myocarditis and cardiac fibrosis in a murine model of autoimmune myocarditis [33]. However, the role of IL-10 in the development of fibrosis during inflammatory resolution has not been clearly defined. Marked induction of lung fibrosis was observed in mice following long-term over-expression of IL-10 by fibrocyte recruitment and M2 macrophage activation [34]. Studies revealed that. IL-10 treatment attenuates ventricular remodeling via activation of signal transducers and activators of transcription (STAT) 3 signaling [35] and inhibition of nuclear factor-κB [36]. Phosphadidylinositol 3-kinase (PI-3-kinase), extracellular signal-regulated kinases (ERK) and JNK pathways are all involved in the dramatic enhancement of IL-10 and alleviation of liver fibrogenesis by glycyrrhizin treatment [37]. Depression of cardiac ERK-mitogen-activated protein kinases (MAPKs) activity is closely related with reduced cardiac fibroblast survival, which in turn alleviates fibrosis, remodelling and cardiac dysfunction [38]. It thus can be speculated that IL-10 might alleviate cardiac fibrosis progression via regulation of Treg and other CD4+ T cell response in ERK, PI3K/AKT and STAT3-dependent pathways. The molecular mechanism of Treg-mediated resolution of CVB3-induced cardiac fibrosis needs further exploration.

In conclusion, this is the first report of a protective role of Tregs in CVB3-induced cardiac fibrosis by secretion of IL-10, suggesting a potential of Tregs in control of adverse ventricular remodeling and cardiac fibrogenesis. This finding would be helpful for the further elucidation of mechanism underlying cardiac fibrosis development and for the exploitation of Tregs as an option for the future design of therapeutic approaches for treating cardiac fibrosis caused by chronic viral infection.
